# Supplementary material for: Discordance between hyposalivation and xerostomia among community-dwelling older adults in Japan
Source: PLoS One. 2023 Mar 3;18(3):e0282740. doi: 10.1371/journal.pone.0282740 (PMC9983907; doi:10.1371/journal.pone.0282740)
Supplement: Attachment — Submitted filename: Response to Reviewers.docx [file pone.0282740.s002.docx]

**Response to editors**

**1. Please ensure that your manuscript meets PLOS ONE's style requirements, including those for file naming.**

The “Statement of Ethics,” “Conflicts of Interest,” “Funding Sources,” and “Author Contribution” statements were deleted from the manuscript file. Furthermore, supporting information text was added. The name of the supporting information files was changed from “Dry Mouth Morita dataset.xlsx” to “S1 Dataset.xlsx”. In addition, a “.” was added after the number for the references list.

**2. We note that the grant information you provided in the ‘Funding Information’ and ‘Financial Disclosure’ sections do not match.**

**When you resubmit, please ensure that you provide the correct grant numbers for the awards you received for your study in the ‘Funding Information’ section.**

The “Funding Information” section was updated to include the grant number.

The Japanese Red Cross Toyota College of Nursing distributes funding for research on an annual basis without grant numbers to full-time researchers. As a grant number is unavailable for this funding source, please state whether it should be removed from these sections.

**3. Please state what role the funders took in the study. If the funders had no role, please state: "The funders had no role in study design, data collection and analysis, decision to publish, or preparation of the manuscript." If this statement is not correct you must amend it as needed.**

The “Financial Disclosure” section previously stated the following:

“The data for this study were obtained from a survey conducted by the Japanese Ministry of Health, Labor, and Welfare's subvention of the medical system for the elderly. This work was supported by the Japanese Red Cross Toyota College of Nursing Fund for Research Support, and the funding was received by Professor I Morita.”

Unfortunately, we erroneously listed the source of origin for the data for this study. Herein denotes the corrected sentence. As you pointed out the funders did not play a role in the study design, data collection, analysis, publication decision manuscript preparation.

“The data for this study were obtained from a survey conducted by the Public Interest Incorporated Foundation 8020 Promotion Foundation Dental Health Activity Project in 2018 (No.9, YA). This work was supported by the Japanese Red Cross Toyota College of Nursing Fund for Research Support, and the funding was received by I M. The funders had no role in study design, data collection and analysis, decision to publish, or preparation of the manuscript.”

Please use this information to update the “Financial Disclosure” section appropriately.

**4. In your Data Availability statement, you have not specified where the minimal data set underlying the results described in your manuscript can be found. PLOS defines a study's minimal data set as the underlying data used to reach the conclusions drawn in the manuscript and any additional data required to replicate the reported study findings in their entirety.**

The following sentence was added to the "Data Availability Statement" section:
 “All data are contained within the manuscript and/or Supporting Information files.”

**5. Please include captions for your Supporting Information files at the end of your manuscript and update any in-text citations to match accordingly.**

Supporting information was added to the manuscript. P11L355

6. Please review your reference list to ensure that it is complete and correct. If you have cited papers that have been retracted, please include the rationale for doing so in the manuscript text, or remove these references and replace them with relevant current references. Any changes to the reference list should be mentioned in the rebuttal letter that accompanies your revised manuscript. If you need to cite a retracted article, indicate the article’s retracted status in the References list and also include a citation and full reference for the retraction notice.

We were unable to identify retracted papers in our reference list. If we have erred in this, please provide the reference number for the retracted article.

Even so, we identified an erratum in reference No. 22 (Turner MD, Ship JA. Dry mouth and its effects on the oral health of the elderly J Am Dent Assoc. 2007). This erratum adds a conflict of interest.

<https://www.sciencedirect.com/science/article/pii/S0002817714614318>

-------------------------------------------------------------------------------------------------------------------------

CORRECTION

Recent supplements to The Journal of the American Dental Association did not contain disclosure notices regarding conflicts of interest held by the authors. Those notices appear below.

| AUTHOR | ARTICLE (PAGE NOS.) | DISCLOSURE REPORTED |
| --- | --- | --- |
| *snip* | | |
| Michael D. Turner, DDS, MD  Jonathan A. Ship, DMD, FDS RCS (Edin) | Dry Mouth and Its Effects on the Oral Health of Elderly People (15S-20S) | None reported  None reported |

-------------------------------------------------------------------------------------------------------------------------

We failed to identify an issue with the description provided. Therefore, we request permission to continue using this reference for our study.

In addition, we have included the following sentences to reference No 22.

“This article includes a conflict of interest erratum. https://www.sciencedirect.com/science/article/pii/S0002817714614318”

We deleted the publication month from the references list.

**Response to reviewer 1**

**in my opinion, this study was done well ,so it was better to use more exact method for assessment of xerostomia.**

**How to use saxon test?**

Thank you for this suggestion. As you highlighted, the Saxon Test does not have high reliability or validity.

As a consequence, we added the following sentence to the discussion.

Revised, P9L251: Furthermore, the Saxon test measurement method does not have very high reliability or validity. Using measurement methods with higher reliability for the stimulated salivary flow rate would provide more precise results.

**it was not clear about sample size?**

We concur that obtaining a sufficient sample size for this study is important. However, because the study was a secondary analysis, sample size calculation was not performed.

**exclusion criteria or inclusion criteria ?**

The inclusion criteria for the studies from which data were collected included: community-dwelling, independent, age 70 years or older and informed consent to participate in the study.

The following sentence was added to the study participants section:

Revised, P3L90: The inclusion criteria were as follows: community-dwelling, independent, age 70 years or older and provided informed consent to participate in the study.

**Response to reviewer 2**

**Overall, a very well-done study; several comments/edits:**

**1. I'm concerned about the question: "Do you worry about dry mouth"; perhaps "Are you experiencing dry mouth" might have led to more accurate answers**

Thank you for this suggestion. Since the original question is in Japanese, its precise translation in English is difficult. Even so, it is quite possible that some participants interpreted the question to mean “Are you experiencing dry mouth?” as you suggested. However, “Do you worry about having a dry mouth?” is a closer translation of the original question. We will keep your comments in mind for future research.

We changed the sentence of question.

Old: Do you worry about dry mouth?

Revised, P3L96: Do you worry about having a dry mouth?

Revised, P6Table 2: Do you worry about having a dry mouth?

**2. I would change the wording on a trend toward age as a factor here as the OR is essentially at the null, and for a cross-sectional study, I think one should say age is NOT a factor in the abstract/results sections.**

Thank you for this suggestion. As you highlighted, this was a cross-sectional study.

We have added the following sentences to the discussion.

Revised, P8L207: As this is a cross-sectional study, the results do not accurately demonstrate the effects of aging. Therefore, the results of this study were unable to provide a clear demonstration of the effect of aging on the dryness of the mouth.

The following sentences were deleted from the discussion.

“This study consolidates that aging may increase the rate of dry mouth, even in the elderly population.”

We have revised the manuscript to replace the word "aging" with "factor of age” in the results section. The changes made to the text are as follows:

Old manuscript: “Aging was significantly associated (OR = 1.105, 95% CI = 1.010 - 1.209) with low SSFR and xerostomia.”

Revised, P2L51: “Age was a factor that was also significantly associated (OR = 1.105, 95% CI = 1.010 - 1.209) with low SSFR and xerostomia.”

Old manuscript: This was also true for aging (OR = 1.105, 95% CI = 1.010-1.209).

Revised, P7L178: This was also true for the factor of age (OR = 1.105, 95% CI = 1.010-1.209).
