# Supplementary material for: Discordance between hyposalivation and xerostomia among community-dwelling older adults in Japan
Source: PLoS One. 2023 Mar 3;18(3):e0282740. doi: 10.1371/journal.pone.0282740 (PMC9983907; doi:10.1371/journal.pone.0282740)
Supplement: Attachment — Submitted filename: Response to Reviewers.docx [file pone.0282740.s003.docx]

Response to Reviewer 3

1- Despite the correction of the inclusion criteria in the first revision, the exclusion criteria have not been determined yet. Please include your exclusion criteria. (it can be for example: having a hearing or language impairment that could affect the interview or having a physical or mental disability that could interfere with the oral exam or …)

Thank you for your suggestion. We added the following sentence to the Study Participants section:

Revised, P3L80:

The exclusion criteria were as follows: hearing or language impairment that could affect the interview, physical or mental disability that could interfere with the oral exam, symptoms, or need for treatment for dental disease.

2- Is there any more appropriate method to measure USFR or SSFR?

This study used visual inspection of the oral cavity by a dentist to measure the unstimulated salivary flow rate (USFR), and the Saxon test was used to measure the stimulated salivary flow rate (SSFR).

As a method of measuring USFR, in addition to the method used in this study, there is a method of collecting saliva. Saliva discharged into the oral cavity in a resting state is exhaled or aspirated, collected, and weighed. Saliva discharged into the oral cavity in a resting state is collected, that is, voided or suctioned and weighed (Tomson, 2015: Ref. no. 1). These methods have the advantage of being highly objective, but stimulation of saliva collection is added. SSFR can be measured using the sense of taste, that is, stimulation by citric acid solution, in addition to stimulation by mastication. Each method has its advantages and disadvantages. Because saliva measurements were performed by a clinical dentist, a method that could be performed by a clinician was adopted.

We added the following sentence to the Discussion section.

Revised, P10L266: Although simple, in this study an appropriate method was adopted for the clinician performing the measurements as the method to measure USFR and SSFR.

3- It is better to consider about exposures and behavioral factors that can effect on xerostomia or hyposalivation such as Condition of dentures, if any, and how to use them, oral hygiene condition, Smoking, history of radiotherapy or chemotherapy and the level of anxiety or stress and etc. Considering that the current study is a secondary analysis based on primary data, was there any information about these factors in the primary data?

The primary dataset did not include other information such as oral hygiene conditions, smoking, history of radiotherapy or chemotherapy, and level of anxiety or stress. Information on the number of teeth and dentures used was included in the primary dataset. However, this information was considered age-related and was excluded from the analysis plan. In addition, due to the limited amount of data available, we did not include information on the number of teeth and dentures used to limit the number of explanatory variables for the multinomial logistic regression analysis.

We added the following sentence to the Discussion section.

Revised, P9L251: This study did not examine exposure and behavioral factors that can affect hyposalivation or xerostomia, such as denture conditions, oral hygiene conditions, smoking, history of radiotherapy or chemotherapy, and level of anxiety or stress.

4- It is also preferable to cite recent sources that were published less than 5 years ago.

Best wishes.

This comment provided us with an opportunity to look through the current literature again. Recognizing that many new papers on dry mouth have been published since we finished writing this paper, we reconfirmed the importance of research in this field. However, with many broad-based reports, it was difficult to determine which additional information would be desirable to add to our paper. We would appreciate if you could specify the information we should cite in our paper.

Response to Reviewer 4

1. The study was well done, but there is a concern and it is the time of saliva sampling. Considering the effect of circadian rhythm on salivary flow, if it is not considered, should be discussed as a limitation of the study.

Saliva flow is known to be affected by the sampling time of the day. However, in the data collection used in this study, it was difficult to measure at the same time of day. Therefore, regarding the concern that saliva sampling was not performed at the same time of day, we added the following to the limitations of the study.

Revised, P9L254: Moreover, salivary flow rate is known to show circadian rhythm and is affected by the sampling time of the day [27]. In the data collection used in this study, measurements were not taken at fixed times of the day and salivary flow rate might have been affected by circadian rhythms. Inconsistency at the time of saliva collection may also be one of the factors making the results unclear.

One reference was added.

27 Dawes C. Circadian rhythms in human salivary flow rate and composition. J Physiol. 1972;220(3):529-245.

2. There are some specific questionnaires for the diagnosis of xerostomia, please add a reference to your mentioned question that was used as a diagnosis tool.

The question in this study used to ask about subjective dry mouth is recommended by the Japanese Ministry of Health, Labor, and Welfare as one of the questionnaire items to evaluate oral function. This question appears to belong to the lineage of questions in Tomson et al. (2007, 2011) and Ohara et al. (2016). These studies were added to the references for the subjective question of dry mouth.

Revised, P3L87: The presence of xerostomia was assessed with one question: "Do you worry about having a dry mouth?" [14-16]. Answer options were either yes or no.

The following references were added.

14 Thomson WM. Measuring change in dry-mouth symptoms over time using the Xerostomia Inventory. Gerodontology. 2007;24(1):30-35.

15 Thomson WM, van der Putten der, de Baat Baat, Ikebe K, Matsuda K, Enoki K, et al. Shortening the xerostomia inventory. Oral Surg Oral Med Oral Pathol Oral Radiol Endod. 2011;112(3):322-327.

16 Ohara Y, Hirano H, Yoshida H, Obuchi S, Ihara K, Fujiwara Y, et al. Prevalence and factors associated with xerostomia and hyposalivation among community-dwelling older people in Japan. Gerodontology. 2016;33(1):20-27.
